# Genome sequence of bacteriophage PensacolaC28 isolated using *Microbacterium sp*. Casco Bay

**DOI:** 10.1128/mra.01146-24

**Published:** 2025-02-20

**Authors:** Lucas R. Girard, Samuel R. Cousins, Alexis C. Heald, Doxel A. Tanzey, Elizabeth M. Omo, Connor R. Flannigan, Hui-Min Chung, Brian P. Tarbox, Emily F. Savage

**Affiliations:** 1 Southern Maine Community College, South Portland, Maine, USA; 2 University of West Florida, Pensacola, Florida, USA; Portland State University, Portland, Oregon, USA

**Keywords:** SEA-PHAGES, HHMI, bacteriophages

## Abstract

Bacteriophage PensacolaC28 is a lytic phage isolated on *Microbacterium sp*. Casco Bay (NCMA B81), a marine bacterium originally cultured in South Portland, Maine, USA. PensacolaC28 was isolated from an environmental sample collected in Pensacola, Florida, USA. It is a singleton siphovirus with a 16,749 bp genome and contains 24 protein coding genes.

## ANNOUNCEMENT

*Microbacterium sp*. Casco Bay is a Gram-positive rod bacterium isolated from marine mud in shallow waters of western Casco Bay, Maine, in March 2020 (Global Positioning System [GPS] 43.6506 N, −70.2318 W). 16s rRNA gene sequencing categorized *M. sp*. Casco Bay as a microbacterial strain; the whole genome has not been sequenced. *M. sp*. Casco Bay is halotolerant and grows on various common growth media, including high salinity and low nutrient agar (1).

**Fig 1:**
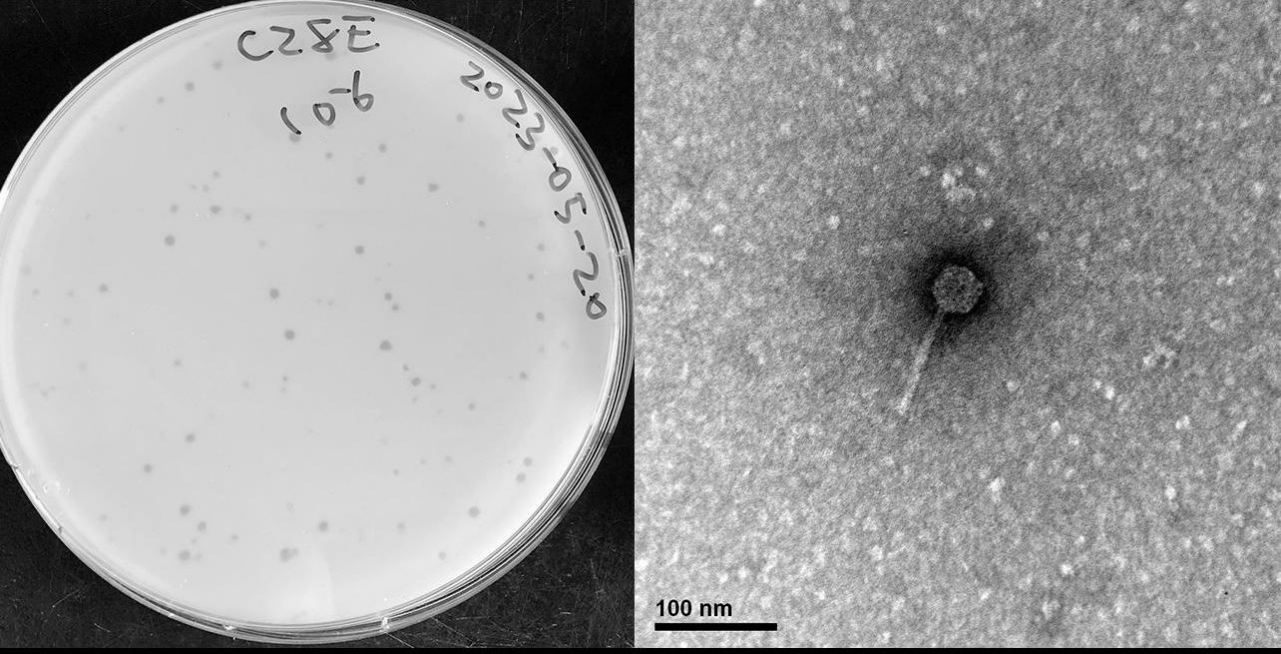
(Left) Plaques (2–3 mm) formed on *M. sp*. Casco Bay by PensacolaC28 after 24 h incubation period at 25°C on PYCa media. (Right) Virion of phage PensacolaC28 was imaged by placing lysate on a Formvar-coated copper grid and then negative stained with 1% uranyl acetate before being imaged using a Hitachi H-7650 microscope operated at 75 kV and equipped with a 1kx1k CCD detection camera (Gatan 782). PensacolaC28 has a tail of 90 nm and an isometric capsid of 36 nm in diameter (*n* = 1).

PensacolaC28 is a bacteriophage isolated from leaf detritus on Navarre beach in Pensacola Florida at the University of West Florida ([GPS] 30.3809 N, −86.85439 W) on 12 March 2023. The sample was collected and isolated using standard protocols (2). A 5 g sample was suspended in 30 mL PYCa (peptone–yeast extract–calcium) liquid medium for 2 days, and the supernatant was then filtered (0.22 µM). Viral particles in the filtrate were then precipitated with PEG 1000 and then resuspended in 1.8 mL phage buffer before being plated in top agar with *Microbacterium sp.* Casco Bay and incubated at 25°C for 24 h, yielding plaques of bacteriophage PensacolaC28 which was purified through three additional rounds of plating. Plaques are 2–3 mm (*n* = 5) in diameter on average with a clear round morphology (Fig. 1). Siphovirus morphology for the virion was determined through negative stain transmission electron microscopy (Fig. 1).

DNA was extracted from a lysate using the Norgen Phage DNA Isolation Kit. Library preparation was done with the NEB Ultra II library kit, and sequencing was conducted on the Illumina MiSeq sequencer using v3 reagents. Sequencing yielded 2,198,872 150-base single-end reads, which resulted in 19,692-fold coverage. Raw reads were assembled and checked by Newbler version 2.9 (3) and Consed V29 (4) using default parameters. The PensacolaC28 genome was 16,749 bp with a 3′ single-stranded overhang. The genome has a 67.2% G + C content. PensacolaC28 did not fit into any known clusters based on gene content similarity of 35% or higher and was classified as a singleton (5) in the Actinobacteriophage database (https://phagesdb.org/) (6).

The genome was annotated using DNA Master (V5.23.6, Build 2705 24 Oct 2021) and PECAAN (https://discover.kbrinsgd.org). Glimmer (v3.02b) (7) and GeneMark (PS-v1.2) (8) were used to identify protein-coding genes. Phamerator (Actino_Draft V561) (9), Starterator (v3.02) (10), Blastp (Actinobacteriophage proteins, non-redundant protein sequences [nr]) (11), and HHPred (PDB_mmCIF70, SCOPe70, Pfam-A, NCBI_Conserved_Domains [CD]) (12) were used to determine gene functions. Aragorn (v1.2.41.c.) (13) was used to confirm the absence of tRNAs, and DeepTMHMM (v1.2.33.c.) (14) and SOSUI (v1.11) (15) were used to detect transmembrane domains. All software was used with default settings.

The genome of PensacolaC28 contains 24 putative protein-coding genes, 19 of which were assigned putative functions, including seven genes for which no homologs exist in phagesDB. Gp8 (F6479-8161) was assigned as a tape measure protein and has only one homolog that is present in phage BigBoyz (Cluster GH) (https://phagesdb.org/genes/BigBoyz_CDS_9/), which was isolated using *M. foliorum*. Gp19 (R14007-14318) is a homolog of gp2 in phage Bluefeather (Cluster FE) (NC_073598), which infects *Arthrobacter globiformis* B-2979 and highlights the pervasiveness of horizontal gene among bacteriophages (16).

## Data Availability

Sequencing results for PensacolaC28 are available in GenBank with accession no. PP978844.1 and Sequence Read Archive (SRA) accession no. SRX25999124. The host strain *Microbacterium sp*. Casco Bay is archived in the National Center for Marine Algae and Microbiota (NCMA) at Bigelow Laboratory for Ocean Science (B81).
